# Host‐parasite dynamics in Chagas disease from systemic to hyper‐local scales

**DOI:** 10.1111/pim.12786

**Published:** 2020-09-24

**Authors:** Damián Pérez‐Mazliah, Alexander I. Ward, Michael D. Lewis

**Affiliations:** ^1^ York Biomedical Research Institute Hull York Medical School University of York York UK; ^2^ Department of Infection Biology Faculty of Infectious and Tropical Diseases London School of Hygiene and Tropical Medicine London UK

**Keywords:** cell mediated immunity, Chagas disease, humoral immunity, in vivo imaging, innate immunity, trypanosomes

## Abstract

*Trypanosoma cruzi* is a remarkably versatile parasite. It can parasitize almost any nucleated cell type and naturally infects hundreds of mammal species across much of the Americas. In humans, it is the cause of Chagas disease, a set of mainly chronic conditions predominantly affecting the heart and gastrointestinal tract, which can progress to become life threatening. Yet around two thirds of infected people are long‐term asymptomatic carriers. Clinical outcomes depend on many factors, but the central determinant is the nature of the host‐parasite interactions that play out over the years of chronic infection in diverse tissue environments. In this review, we aim to integrate recent developments in the understanding of the spatial and temporal dynamics of *T. cruzi* infections with established and emerging concepts in host immune responses in the corresponding phases and tissues.

## 1 *TRYPANOSOMA CRUZI*: A FORMIDABLE FOE

*Trypanosoma cruzi*, the causative agent of Chagas disease (American trypanosomiasis), is an extraordinarily versatile parasite. Its wild transmission cycles across the Americas are maintained by over 100 species of haematophagous triatomine bugs. Chagas disease is a zoonosis, and *T. cruzi* infects diverse mammal reservoir species, including marsupials, bats, rodents, ungulates, carnivores (including domestic cats and dogs), armadillos, pilosans and primates.^1^ *T. cruzi* undergoes regulated morphological transitions involving at least four developmental forms, each with a distinctive biology, for example cell structural features, modes of motility, surface protein coats^2^ and metabolic programmes.^3^ The epimastigote form replicates in the vector's gut, then differentiates to a highly motile form, the metacyclic trypomastigote, which invades mammalian host cells after transmission. Potentially any nucleated cell type may be parasitized in any tissue the trypomastigote can reach.^4^, ^5^, ^6^, ^7^, ^8^, ^9^, ^10^, ^11^, ^12^, ^13^, ^14^, ^15^, ^16^ After invasion and escape from a parasitophorous vacuole into the cytosol, another transition occurs to the amastigote form, which replicates repeatedly and then differentiates to generate a population of pleomorphic tissue/bloodstream form trypomastigotes. These are released into the extracellular space, from where they may infect a new cell, in some cases after migration to the bloodstream. Alternatively, in the event they are taken up in a triatomine blood meal, they can complete the cycle by differentiating into epimastigotes.

Mounting evidence shows *T. cruzi's* life cycle is considerably more complex than the textbook view. Findings include epimastigote‐like forms in mammals, asynchronous replication and trypomastigogenesis, asymmetric divisions, reversible transitions and formation of apparently quiescent or dormant amastigotes.^17^, ^18^, ^19^, ^20^, ^21^, ^22^ The morphological, antigenic and spatial variability, combined with active evasion strategies, presents a formidable challenge to the mammalian immune system. Nevertheless, most infections resolve to a stable chronic equilibrium of parasite replication and suppression via a combination of sustained antibody and type 1 cellular responses. The majority of people (>95%) survive acute infection and progress to a chronic, asymptomatic phase. Chagas cardiomyopathy is then estimated to develop at a rate of ~2% per year.^23^ Disorders of the gastrointestinal (GI) tract develop in a smaller proportion of cases, sometimes in combination with cardiac disease.^24^ Why Chagas pathology only affects a limited subset of tissues, in only a specific subset of infected people, is one of the longest‐standing and most important unanswered questions in the field.

In this review, our aim is to integrate recent developments in the understanding of the spatial and temporal dynamics of *T. cruzi* infections with established and emerging concepts in host immune responses in the corresponding phases and tissues. The result is a view that parasite persistence occurs in a small number of privileged tissues alongside highly competent, *T. cruzi*‐specific systemic responses, suggesting a substantial degree of compartmentalization, even within tissues. The low‐level, yet perpetual chronic inflammation has the potential to become pathological, dependent on largely undefined host, parasite and environmental factors. Thus, progress in the development of anti‐parasitic drugs, adjunct treatments, immunotherapies and vaccines is likely to require a much better understanding of the molecular and cellular determinants of *T. cruzi* persistence at the tissue‐specific and even hyper‐local, intra‐tissue scale.

## 2 ADVANCES IN STUDIES OF TISSUE‐SPECIFIC INFECTION DYNAMICS

**FIGURE 1:**
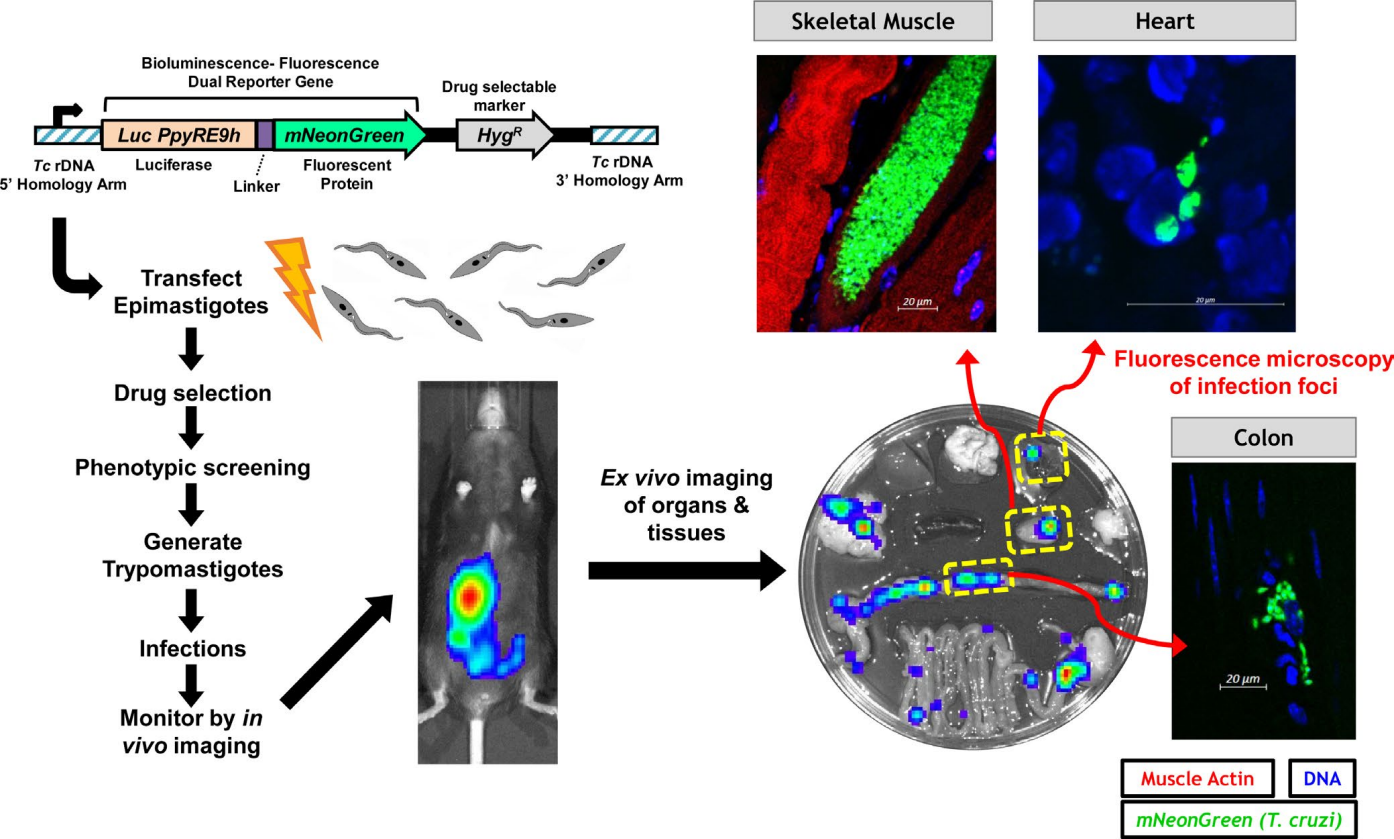
Visualization of *Trypanosoma cruzi* infection foci in vivo using bioluminescent‐fluorescent dual reporter parasites

While *T. cruzi* must spend some time in extracellular environments of the blood and interstitial fluid to sustain infections and ensure transmission, it is predominantly an intracellular parasite of solid organs. Consequently, most of what is known of the cells and tissues targeted by *T. cruzi* comes from experimental animal studies. It is difficult to obtain robust data on tissue distribution in human patients, although post‐mortem, transplant and biopsy results tend to be consistent with animal models. The mouse is the species of choice, but other rodents, rabbits, dogs and nonhuman primates have also demonstrated utility.^25^ Tissue‐specific parasite loads can be measured by a range of direct and indirect methods (reviewed in 26). Developments in real‐time bioluminescence imaging methods have underpinned much recent progress in understanding *T. cruzi* infection dynamics.^4^, ^5^, ^27^, ^28^, ^29^, ^30^, ^31^ These systems are based on transgenic parasites expressing luciferases, enabling analysis of light signals emitted by parasites in discrete anatomical locations. Major advantages include greatly reduced tissue sampling bias and the ability to monitor individual mice over time. Bioluminescence lacks the resolution necessary to visualize parasites at individual cell scale, but this can be achieved using parasites expressing fluorescent reporters,^21^, ^32^, ^33^ an approach that becomes particularly powerful when luciferase‐fluorescence fusion proteins are employed (Figure 1).^22^, ^34^ The possibility to integrate these imaging methods with analyses of concomitant immune responses^35^ holds considerable promise for advancing our understanding of *T. cruzi*‐ host interactions.

## 3 STAGE 1: *T. CRUZI* SYSTEMIC COLONIZATION AND INNATE RESPONSES

In vectorial transmission scenarios, infection results from contamination of the triatomine bite wound or of mucosal membranes with trypomastigotes present in the bug's faeces. Transmission may also occur orally, via contaminated food or drink, *in utero*, and by blood transfusion and organ transplant. Several mechanisms of host cell invasion have been described and reviewed elsewhere.^36^ In humans, oedema with intense mononuclear infiltrate at the entry site in the skin (chagoma) or eye (Romaña's sign) indicates an initially very localized infection.^37^ However, the true extent of trypomastigote dissemination is not clear and surprisingly little is known at the cellular level about the actual sites of primary invasion and the first cycle of intracellular parasite replication, which lasts approximately 1 week. Experimental animal studies indicate that the route of inoculation is a key factor. Intra‐peritoneal injection results in similar parasite numbers in diverse tissues after 6 days.^38^ Conversely, oral transmission results in highly localized infections in the stomach or nasomaxillary tissues^31^, ^38^, ^39^ with initial infection of the local mucosal epithelium.^40^ Similarly, after conjunctival inoculation, parasites first invaded and replicated in the mucosal epithelium of the nasolacrimal ducts and nasal cavities.^41^

At the end of the first intracellular cycle, trypomastigotes are released and the infection disseminates widely. *T. cruzi* is pan‐tropic in the acute phase of infection (reviewed in 26). However, the relative intensity of infection in different cell or tissue types again varies depending on the inoculation route and inoculum size, as well as intrinsic factors such as replication rate and capacity for dissemination. Sites reported to harbour the highest acute infection intensities include skeletal, smooth and cardiac muscle and adipose tissues. Some studies have described *T. cruzi* strains with an increased capacity to parasitize mononuclear phagocytes^13^, ^42^, ^43^ or to cross the blood‐brain barrier.^12^, ^44^, ^45^

### 3.1 Sensors

The host response to *T. cruzi* primary infection is considered to be markedly delayed by comparison with model intra‐cytosolic pathogens.^46^, ^47^ The main features of the immediate response are the induction of type I interferon signalling, and recruitment of neutrophils, macrophages and Natural Killer (NK) cells.^48^ Ca^2+^ mobilization, associated with invasion of myeloid cells, can activate the transcription factor NFATc1, leading to interferon gamma (IFNγ) production by NK cells and dendritic cell (DC) maturation.^49^ *T. cruzi* also produces multiple B‐cell mitogens that directly trigger a robust T‐independent B‐cell activation.^50^, ^51^, ^52^

Few canonical pathogen‐associated molecular patterns (PAMPs) are conserved in *T. cruzi*. The best characterized innate pattern recognition receptors (PRRs) for *T. cruzi* PAMPS are Toll‐like receptor (TLR) 2 and 9. These recognize, respectively, the glycophosphatidylinositol (GPI) anchor of parasite surface proteins and parasite DNA, specifically unmethylated CpG motifs.^53^, ^54^ TLR2 + 9 double‐knockout mutant mice suffer higher parasitaemias and significantly increased mortality rates (50% by day 50) compared to wild‐type controls.^54^ Mice lacking both MyD88 and TRIF, thus rendered incapable of any TLR‐mediated responses, have uncontrolled parasitaemia and 100% mortality by 18 days of infection.^55^ This may be explained by the additional involvement of TLR4 and TLR7, recognizing parasite glycoinositolphospholipids and RNA, respectively.^56^, ^57^, ^58^

Many *T. cruzi* surface proteins are extensively glycosylated,^2^ and several host galectins (a widely expressed family of carbohydrate‐binding proteins) are able to bind them.^59^ Interactions involving several different galectins may actually help *T. cruzi* bind to and enter host tissues,^60^, ^61^ but this does not appear to directly trigger any anti‐parasitic effector activity. As an occupant of the host cell cytoplasm, it is likely that *T. cruzi* triggers cytosolic sensors. The best known candidate systems centre on NOD‐like receptors (NLR). Mice lacking the NOD1 receptor suffer 100% mortality to acute *T. cruzi* infection, although the mechanism explaining this remains obscure.^62^ Studies also support a parasite‐suppressive role for the related receptor, NLRP3 and downstream components of the inflammasome complex that drives IL‐1β and IL‐18 secretion,^63^, ^64^, ^65^ although, as with NOD1, it is not clear if this involves direct sensing of *T. cruzi* in vivo.

The majority of studies of innate immunity to *T. cruzi* have focussed on responses in myeloid cells, especially macrophages, yet these could represent only a minor subset of the parasite's early targets. Transcriptomic analysis of in vitro*‐*infected human fibroblasts revealed that inflammatory cytokine expression peaked 24 hours post‐infection and the TLR‐independent type I IFN response became the dominant signature by 72 hours, which was suggested to promote, rather than inhibit, the infection.^66^ Trypomastigotes have a diverse secretome comprising proteins in native form and also as cargoes in shed extracellular vesicles.^67^, ^68^, ^69^, ^70^ Important research questions to address include whether and at what point these are relevant for triggering PRRs in vivo and whether there are equivalent processes for intracellular amastigotes.

After the first cycle of replication ends, host cells rupture and trypomastigotes escape into the extracellular environment. At this point, trypomastigotes may invade local tissue cells, enter infiltrating leucocytes or migrate via the blood or lymphatics to other tissues. The factors governing the parasite's propensity to stay local or not remain obscure. Host cell rupture releases intracellular material rich in danger‐associated molecular patterns (DAMPs), further stimulating innate signalling via TLRs as well as, for example, degranulation of nearby mast cells and activation of myeloid cells. Recently, sensing of oxidized DNA in extracellular vesicles via cyclic GMP‐AMP Synthase (cGAS) was identified as an important DAMP recognition mechanism for macrophage activation.^71^ As a eukaryote, *T. cruzi* has endogenous orthologues of many mammalian DAMPs, potentially blurring the boundaries between DAMPs and PAMPs. For example, recombinant *T. cruzi* High Mobility Group B (TcHMGB) protein can induce production of nitric oxide (NO) in macrophages in vitro and expression of genes encoding inflammatory cytokine genes in vivo.^72^

### 3.2 Signal mediators and amplifiers

A plethora of cross‐talking signalling pathways are activated downstream of the PAMP/DAMP sensors described above. Signalling converges on a set of transcription factors (NF‐κβ, AP‐1, IRF3), which results in production of inflammatory cytokines.^73^, ^74^, ^75^, ^76^ Critical amongst these are the IL‐12 family, IFNγ and TNF‐α, the canonical drivers of type 1 immune responses required to tackle intracellular infections. IL‐12 is essential for the early activation of recruited natural killer cells and their production of IFN‐γ; both these cytokines are indispensable for control of parasite loads and avoidance of acute mortality.^77^ TNF‐α is also essential for survival of the acute stage.^78^ IFNy and TNF‐α activate parasite destructive effector mechanisms via autocrine and paracrine signalling. Beyond the canonical IL‐12‐IFNγ axis, signalling through the IL‐1 receptor is essential for early (10 days p.i.) induction of the myocarditis needed to control heart parasitism.^65^

The local tissue response is amplified via chemokine‐driven recruitment of inflammatory monocytes, macrophages, neutrophils and, eventually, antigen (Ag)‐specific CD4^+^ helper T and CD8^+^ cytotoxic T lymphocytes (Th and CTL) to the site of infection.^79^ Microvascular plasma leakage into parasitized tissues is promoted further by activation of mast cells and the kallikrein‐kinin system (KKS), via a mechanism involving cruzipain, a parasite‐derived cysteine protease.^80^ The resulting tissue oedema and upregulation of associated receptors on cardiomyocytes may increase specific susceptibility to heart invasion as the infection progresses.^81^

### 3.3 Innate effectors and their evasion

The infection, cell necrosis and associated inflammatory signalling result in the activation of a range of innate effector mechanisms. There is some evidence from analysis of Beclin‐1‐deficient mice that host cell autophagy can provide some marginal early restraint on parasite replication.^82^ Epimastigotes are complement‐sensitive but trypomastigotes have effective molecular mechanisms providing resistance to complement‐mediated lysis.^83^ Infiltrating NK cells, in addition to being major producers of IFNγ, may have direct parasiticidal effects involving the release of cytotoxic granules.^84^

An unusual population of innate‐like CD8^+^ T cells with activation characteristics of (e.g. production of granzyme A and IFNγ) expands in the thymus of *T. cruzi* ‐infected mice. These cells appear to be driven by antigen‐independent mechanisms, and adoptive transfer experiments of thymocytes from infected mice suggest they might provide protection from otherwise lethal challenge^85^; however, the underlying mechanisms conferring this protection remain to be elucidated.

Reactive oxygen and nitrogen species (ROS, RNS) are principal effectors for *T. cruzi* control. These are generated by IFNγ/TNF‐α–activated macrophages and via diverse other mechanisms in non‐phagocytes and extracellular compartments.^86^ They are a significant cause of collateral damage in infected tissues, but high levels are necessary because *T. cruzi* has an extensive and highly effective antioxidant defence system.^86^, ^87^ ROS can even promote *T. cruzi* replication, by a mechanism proposed to depend on the increased availability of intracellular Fe^2+^ ions that the parasite can utilize.^88^ Nitric oxide (NO) is directly parasiticidal in vitro,^89^ and inducible NO synthase (iNOS) is essential for in vivo parasite control in some models,^62^, ^77^, ^90^ although not in others.^63^, ^91^

Despite the plethora of innate responses, the overall effectiveness of *T. cruzi's* evasion mechanisms renders it debatable whether they actually have any meaningful impact on most infections, apart from the induction and conditioning of the adaptive response (see below). Indeed, *T. cruzi* infections are 100% lethal in mice that are genetically incapable of mounting adaptive responses (SCID, RAG, nude)^92^, ^93^, ^94^ and bioluminescence imaging studies show that parasite growth in such mice is close to exponential.^4^

## 4 STAGE 2: ADAPTIVE RESPONSES TAKE CONTROL

The infection usually peaks, in terms of total parasite numbers and the extent of tissue dissemination, at a point between 2 and 3 weeks post‐infection. Over the following weeks, parasite loads are reduced by several orders of magnitude by a highly effective adaptive immune response. Although they are ultimately thought to be non‐sterilizing in virtually all cases,^95^ it is worth reviewing the key features at the systemic level before we consider the tissue‐specific host‐parasite dynamics at play in the chronic phase. We also refer readers to more in‐depth reviews of adaptive immunity in Chagas disease.^46^, ^96^, ^97^

### 4.1 T‐ and B‐cell activation

*T. cruzi* cycles between the cytosolic and extracellular compartments and, accordingly, its control is critically dependent on the generation and deployment of Ag‐specific CTL to infected tissues and antibody production by B cells. This is evidenced by relevant gene disruption and antibody‐mediated depletion experiments in mice.^98^, ^99^, ^100^, ^101^, ^102^ Mature DCs in the spleen and lymph nodes draining infected tissues, conditioned by the inflammatory environment, activate parasite Ag‐specific CD8^+^ and CD4^+^ T cells from the naïve pools.^47^, ^103^ Activated T cells then migrate to sites of infection to exert effector mechanisms or, in some cases, begin differentiation to memory subsets.^102^, ^104^ A number of factors may impinge on the quality and magnitude of the T‐cell response, including parasite‐driven immature thymocyte apoptosis^105^ and direct and indirect modulation of DC‐T cell interactions.^106^, ^107^, ^108^, ^109^ In terms of antigen specificity, the murine T‐cell repertoire is focussed mainly on a small number of immunodominant epitopes from highly expressed surface proteins,^110^, ^111^, ^112^ but in humans there is evidence of a broader hierarchy^97^, ^113^ and immunodominance appears not to directly contribute to chronicity.

The role of CD4^+^ T cells is not well characterized, but the association between HIV infection and life‐threatening acute *T. cruzi* relapse in humans^114^ indicates they are critical for parasite control. Accordingly, mice that are specifically incapable of mounting CD4^+^ T‐cell responses experience 100% acute lethality of *T. cruzi* infection.^115^ This has been linked to loss of support for parasite‐specific CD8^+^ T‐cell cytotoxicity against intravenous delivered splenocytes loaded with parasite antigens from the ASP‐2 gene,^99^ but not in similar experiments using trans‐sialidase peptides.^116^ This may reflect differing requirements for T‐cell help depending on immunodominance hierarchies.^97^ Nevertheless, the majority of CD4^+^ T cells develop a protective Th1 profile and contribute further to the abundance of type 1 cytokines, particularly IFNγ.^93^, ^117^, ^118^, ^119^ Broader phenotypic diversity does develop alongside Th1 predominance, including minor Th17, Th1/Th17 intermediate and possibly Th2 subsets in some circumstances.^118^, ^120^, ^121^, ^122^, ^123^, ^124^ There is no clear consensus on the relevance of these to parasite control and immunopathogenesis, but this is an active area of research. The CD4^+^ T cells that provide B‐cell help are termed follicular helper T cells (Tfh), and represent a distinct CD4^+^ T‐cell programme regulated by the master transcription factor Bcl6. Although activation of Tfh responses to *T. cruzi* infection has not been explored in detail, it is reasonable to hypothesize that they are required for the production of parasite‐specific antibodies and ultimate control of the infection. In line with this, IL‐6, which supports Tfh differentiation,^125^, ^126^ is required for the control of parasitaemia and splenocyte recall response to parasite antigens,^127^ but not for T‐cell independent polyclonal activation of B‐cell responses.^51^

The initial B‐cell response in the spleen is estimated to be at least 10‐fold higher as compared to LNs draining infected tissues,^52^ and a robust *T. cruzi*‐specific antibody response is still generated there alongside the aforementioned polyclonal B‐cell activation and non‐specific hyper‐gammaglobulinaemia. The parasite‐specific antibody response is presumably driven by B‐cell activation involving T‐cell collaboration because it is accompanied by a robust germinal centre B‐cell response and production of parasite‐specific class‐switched antibodies.^52^ The specific and non‐specific splenic B‐cell responses appear to be either differentially regulated or carried out by different B‐cell compartments because only the latter depend on the cytokine B‐cell activating factor (BAFF).^128^

Activation of auto‐reactive T‐ and B‐cell clones, the latter leading to the production of autoantibodies, is a well‐described phenomenon during *T. cruzi* infection.^129^ Polyclonal B‐cell activation, host molecular mimicry by parasite proteins and bystander activation caused by tissue damage have been postulated as underlying mechanisms.^129^ There is broad evidence and consensus that parasite persistence is required to sustain these autoimmune responses.^130^, ^131^, ^132^ Nevertheless, the significance of autoantibodies and auto‐reactive T cells for Chagas disease pathogenesis and the mechanisms involved in their production during *T. cruzi* infection remain major unresolved questions.

It has been suggested that the non‐specific polyclonal B‐cell activation contributes to delay the generation of *T. cruzi* ‐specific B‐cell responses, thus contributing to parasite escape and establishment of chronic infections.^133^, ^134^ Polyclonal B‐cell activation is associated with rapid, innate‐like production of IL‐17 and IL‐10^135^, ^136^ but the wider relevance is unclear as both protective^136^, ^137^, ^138^ and deleterious^51^, ^52^, ^135^ roles for such innate‐like B‐cell responses have been documented in different models. The infection also causes a transient, yet marked loss of immature B cells in the bone marrow in experimental mouse models, possibly further compromising the response.^139^

The kinetics of the adaptive response depend to some extent on the early parasite load,^99^ but in most cases, it coincides with the second or third intracellular cycle of parasite replication and is considered relatively delayed.^47^ Nevertheless, substantial immune memory can be generated quite rapidly: mice whose infections were cured by benznidazole anti‐parasitic chemotherapy starting 4 or 14 days after infection were then able to restrict acute parasite loads in challenge infections by 85% and >99%, respectively.^35^ Notably though, very few of these animals achieved sterile cure and they progressed to chronic phase infections that were comparable to primary infections in naïve mice. This raises important questions about what is mediating memory responses to secondary infections, for example whether they are T cell‐dependent or independent.

### 4.2 Adaptive effector mechanisms

Lymphocytes contribute to control of *T. cruzi* by production of type 1 cytokines that amplify the prior, innate ROS and RNS production in infected tissues. Their signature, direct effector mechanisms are also crucial. These include the principal CTL effector pathways, namely perforin‐mediated delivery of granzymes and FasL‐induced apoptosis. In particular, granzymes cause fatal oxidative damage to *T. cruzi*, which can be mitigated by ROS scavenging drugs or overexpression of parasite antioxidant genes.^140^ This may potentially be accelerated in humans by granulysin‐mediated delivery of granzymes directly into intracellular parasites themselves.^140^ Mice do not have a granulysin gene but in most cases still achieve good control of parasite levels, so immune pressure may be more focussed on extracellular amastigotes after host cell apoptosis and on clearance of trypomastigotes. These canonical pathways are essential in some experimental models^140^, ^141^, ^142^ but dispensible in others.^98^, ^143^ The difference is likely explained by other pathways providing sufficient compensatory effector capacity in lower parasite load or virulence scenarios.

*T. cruzi*‐specific lytic and neutralizing antibodies are normally detected in humans and animal models.^144^, ^145^, ^146^, ^147^ These are mostly produced in the spleen; antibody secretion by bone marrow cells obtained from acutely infected mice is below detection level.^52^ This suggests that either plasma cells generated in secondary lymphoid organs during acute *T. cruzi* infection are unable to migrate to the bone marrow, or that the bone marrow may not sustain plasma cell survival during the early phase of the infection, or that plasma cell homing in the bone marrow is somehow delayed during *T. cruzi* infection. Whether this is a temporary mechanism or extends throughout chronic infection and whether it is a direct mechanism driven by presence of the parasite in the bone marrow are unknown.

Antibodies target extracellular trypomastigotes, but they may also have a role in binding amastigotes released from ruptured host cells, for example, downstream of CTL‐mediated lysis. Opsonized parasites are efficiently taken up by tissue‐resident macrophages, especially in highly vascularized organs, for example liver, lung, spleen.^148^, ^149^ It is not clear how a subset of trypomastigotes evade this fate to sustain chronic infections. Beyond their role as antibody producers, B cells are also critically required for functional T‐cell responses to control *T. cruzi* infection ^102^, ^150^, ^151^, ^152^ and production of cytokines, including IL‐17 and IL‐10.^135^, ^136^

### 4.3 Deactivating/Regulatory mechanisms

The strong and sustained systemic inflammation, host cell lysis and tissue parasite killing in this control phase cause potentially dangerous levels of collateral tissue damage. Infections may become overtly symptomatic and in some cases fatal, particularly if the CNS is involved.^153^ Tissue‐protective immune regulatory pathways are therefore initiated to dampen the inflammatory response, to the benefit of the remaining parasites, which form the founding populations of the chronic infection reservoirs (Figure 3).

The factor with the strongest evidence for an important regulatory role is probably the cytokine IL‐10. Early studies of IL‐10 deficiency using high virulence Tulahuen strain parasites reported better control of acute *T. cruzi* parasitaemia at the expense of rapidly fatal (~2 weeks p.i.) pathogenic inflammation, for example TNF‐α–mediated toxic shock.^154^, ^155^ More recent studies point to greater complexity. Rôffe *et al* (2012) reported IL‐10 was essential to protect against later mortality (3‐6 weeks p.i.) associated with poor control of Colombiana strain tissue parasite loads and increased myocarditis intensity. In still lower virulence scenarios, the absence of IL‐10 has been associated with reduced CTL effector function but without any increased mortality.^156^ Both CD8^+^ and CD4^+^ T cells are IL‐10 sources, and a high proportion simultaneously produce IFNγ,^157^ likely supported by IL‐27 production^158^ and potentially in direct response to parasite shed *trans*‐sialidase.^123^ B cells also produce IL‐10,^135^ and overall IL‐10 production is lower in B1 B‐cell‐deficient mice early during infection.^159^ CD11b^+^ B1 B cells from asymptomatic, infected individuals show increased capacity to produce IL‐10 compared to those with cardiac disease symptoms.^138^ In addition, recent data show that when compared to non‐infected donors, chronically *T. cruzi*‐infected individuals with cardiac manifestations have an increased proportion of immature transitional CD24^high^CD38^high^ and naïve B cells able to produce IL‐10 upon in vitro re‐stimulation.^160^ This suggests B cell–intrinsic IL‐10 signalling might be important to regulate the intense adaptive immune response, as is the case for other parasitic infections,^161^ but direct mechanistic evidence is required to support this hypothesis.

Transforming growth factor beta (TGF‐β), another potent regulatory and tissue‐protective cytokine, can be activated from its latent form by a *T. cruzi* protease (cruzipain) in vitro.^162^ TGF‐β signalling to T cells reduces the risk of late acute mortality, and this appears to involve inhibition of cell proliferation rather than suppression of inflammatory cytokine production.^106^, ^163^ Other factors potentially contributing to early inhibition of adaptive immune effector responses include suppressor of cytokine signalling (SOCS),^164^ regulatory CD4^+^ T cells (Tregs)^106^, ^165^ and induction of various regulatory/suppressive myeloid cell phenotypes, such as expression of iNOS‐limiting arginase.^109^, ^166^, ^167^

**FIGURE 2:**
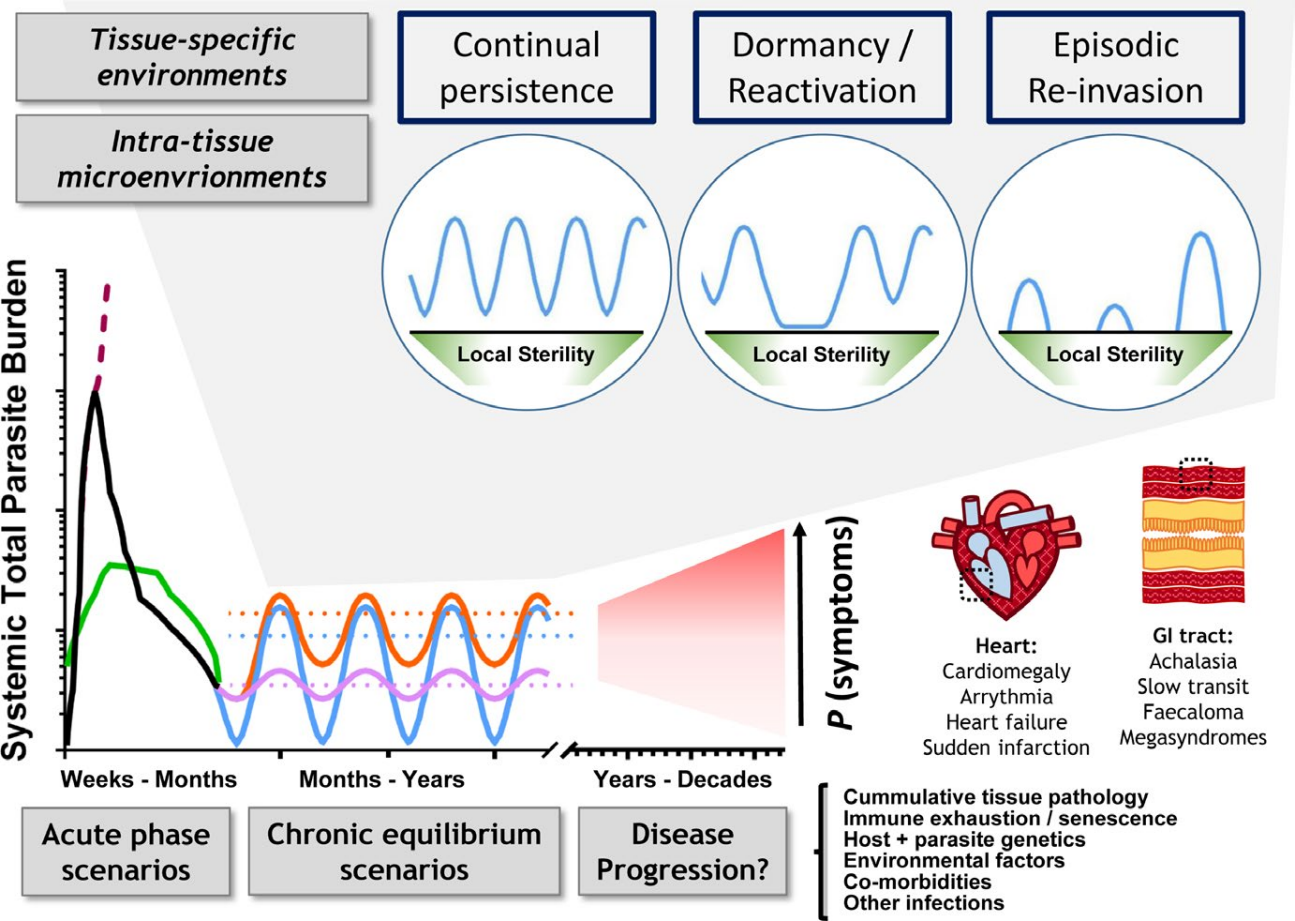
Overview of host‐parasite interaction dynamics in *Trypanosoma cruzi* infections. Chart illustrates some alternative course of infection scenarios for acute and chronic phase total parasite burdens, feeding into potential clinical outcomes, which range from long‐term non‐progression to severe Chagas disease affecting the heart and/or gastrointestinal tract. The chronic equilibrium scenarios are the product of many temporally overlapping host‐parasite interactions within and between the various organs targeted for infection by *T. cruzi*. Three possible, non‐mutually exclusive modes of persistence at the tissue or tissue sub‐domain level are illustrated above the chart, continual persistence, dormancy/reactivation and episodic re‐invasion.

**FIGURE 3:**
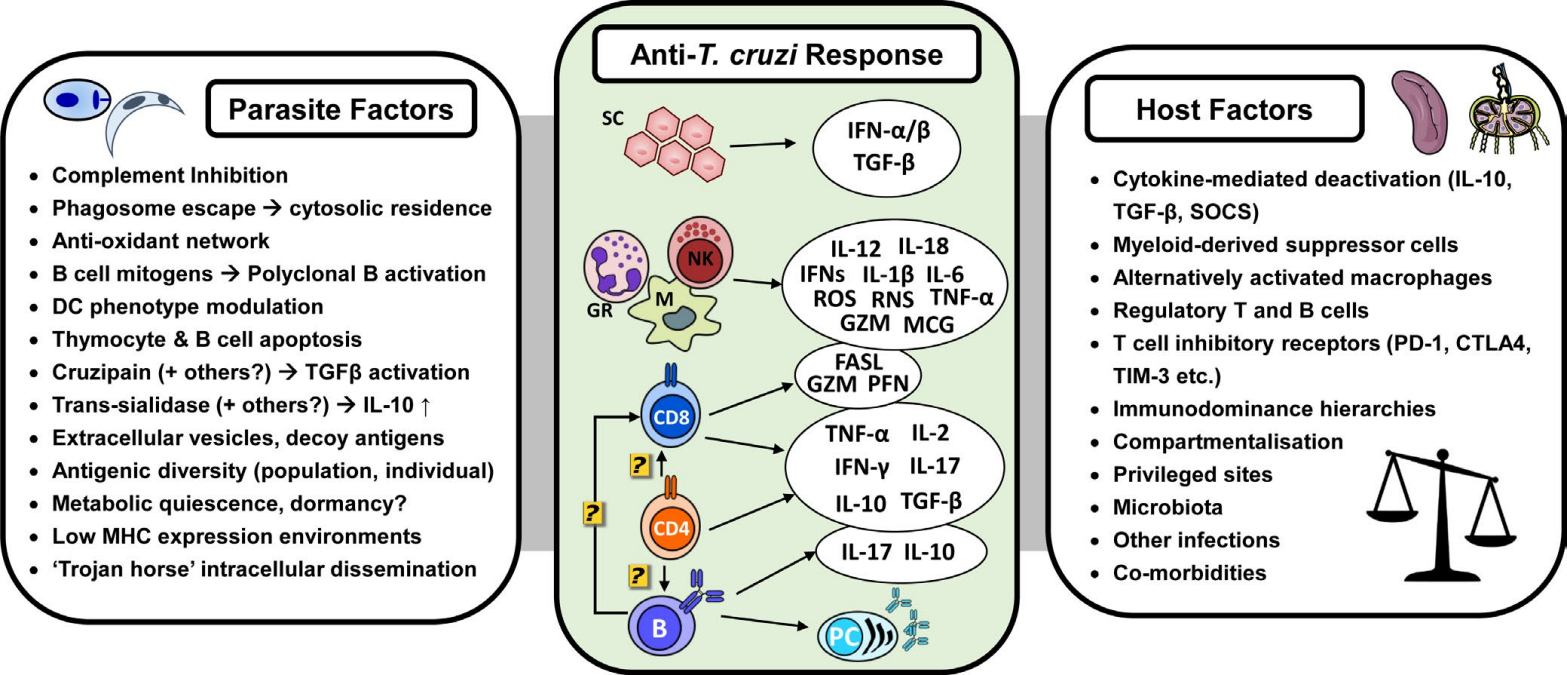
Overview of the principal cellular and molecular components of the immune response to *Trypanosoma cruzi* infection. Central panel shows key cell types, cytokines and effector molecules. B, B cell; CD4, helper T cell; CD8, cytotoxic T cell; GR, granulocytes; M, myeloid cells; NK, natural Killer cells; PC, plasma cell; SC, somatic cells. Left and right panels show parasite and host factors potentially contributing to infection chronicity.

The overall result of these deactivating pathways is the avoidance of potentially life‐threatening levels of inflammation and tissue damage at the expense of incomplete clearance of the infection (Figures 2 and 3). The situation at the tissue‐specific level, however, is likely to be more complex because only a subset of tissues serve as privileged sites for *T. cruzi* persistence in the chronic phase.^26^

## 5 STAGE 3: THE 1% AND THE CHRONIC HOST‐PARASITE EQUILIBRIUM

In the chronic phase, blood parasitaemia is typically sub‐patent and tissue parasite loads are between 0.1% and 1% of their levels in the acute phase.^4^, ^29^ Animal imaging studies^4^ and serial analysis of patient blood by PCR^168^ show infection levels fluctuate over time, pointing to a dynamic equilibrium between intracellular parasite replication, antibody and effector T‐cell activity. The state of this equilibrium at the organismal level is the product of many discrete host‐parasite interactions within and between multiple tissues (Figure 2). Over time, these interactions can become overtly pathological and subsets of infected people develop a spectrum of symptomatic forms of Chagas disease, as reviewed elsewhere.^169^

Given the difficulty in sampling tissue parasites from humans, most of our knowledge on their tissue distribution comes from animal models. These indicate that chronic infection dynamics are shaped by the combination of *T. cruzi* strain and the host's genetic background. It appears that the GI tract, mainly the large intestine and stomach, is a universal site of continual parasite persistence in mice.^29^, ^30^, ^170^ The well‐studied parasite strain CL Brener is also commonly detected in the skin of BALB/c mice^38^ but only sporadically in other sites, for example skeletal muscle, lung, adipose. More virulent parasite strains (eg Brazil, Colombiana, Tulahuen, VFRA) and certain mice (eg C3H) are associated with more disseminated infections, including heart and skeletal muscle localization.^29^, ^30^, ^170^, ^171^, ^172^, ^173^, ^174^, ^175^ There have been few robust data on the relevant cell types within any of these chronically infected tissues, but recent in vivo imaging analysis at single cell resolution revealed smooth muscle cells as the most frequent targets in the colon.^175^

A population of central memory T cells (T_CM_) is detectable during the chronic phase of infection, which may stem from a lack of parasite antigen in lymph nodes draining non‐parasitized organs.^104^, ^176^ These T_CM_ are maintained after benznidazole‐mediated cure of chronic infection and provide protection against re‐challenge after transfer into naïve mice.^177^ Homologous challenge infections, in drug‐cured mice themselves, result in acute parasite loads <1% of those in primary infection controls, and fully sterile protection is seen in around half of the animals.^35^ The determinants of both categories of protection remain to be defined, but it is likely critically dependent on the T_CM_ population. It is likewise an open question whether T_CM_‐derived effectors contribute to suppression of tissue parasite numbers during chronic infections, particularly in organs subject to cycles of episodic re‐invasion and clearance.

The mechanisms of immune evasion sustaining the host‐parasite equilibrium during perpetual chronic infection are not necessarily the same as those that prevent sterile clearance in the acute to chronic transition, which resemble a conserved host tissue‐protective, anti‐inflammatory programme. They are also harder to study, both from the parasite perspective, owing to the scarcity of *T. cruzi* foci in tissues, and from the host perspective because of the need for conditional intervention techniques that can be applied after acute infections have been brought under control. The essentiality of CD8^+^ T cells for continued suppression of parasite numbers in the chronic phase is reasonably clear. Parasite loads rebound rapidly upon treatment with anti‐CD8 antibodies, almost to the level seen with pan‐adaptive immunosuppression using cyclophosphamide,^174^, ^178^ although depletion of CD8^+^ NK cells and DCs may contribute to the relapse, in addition to CTLs. Unlike in the acute phase, experimental anti‐CD4 treatment has no effect on chronic parasite loads.^178^ Nevertheless, severe reactivation of Chagas disease in HIV co‐infected patients indicates CD4^+^ T cells are vital for control and the frequent presentation of meningoencephalitis points to a specific role in protection against invasion of the CNS.^114^

There are various non‐exclusive hypotheses for how a small subpopulation of parasites reliably evades sterilization in the face of the sustained adaptive immune pressure. However, in our view none currently has compelling evidence supporting a mechanistic explanation so this will remain an active area of investigation.

### 5.1 Antigenic diversity

African trypanosomes famously evade host immunity using a system of antigenic variation, involving tightly regulated mono‐allelic expression and switching of variant surface glycoprotein genes,^179^ but this is not conserved in *T. cruzi*. The *T. cruzi* genome contains enormous repetitive arrays of surface protein genes, and there is evidence that some of these gene families or sub‐families are reserved for expression in specific life cycle stages.^2^, ^180^ Signatures of strong positive selection in surface gene families^181^ indicate immune pressure for diversification of antigens. At the population level, simultaneous expression of massively diverse ‘decoy’ antigens may conceivably prevent T‐ or B‐cell clones specific to any particular epitope from reaching sufficient frequency in infected tissues and/or effector capacity,^83^, ^112^, ^180^, ^182^ but direct evidence for this is lacking.

Very little is known about how variant copy expression may be controlled at the individual cell level, that is amongst amastigotes and amongst trypomastigotes. Investigating this is difficult, because gene control is mainly post‐transcriptional and suitable variant‐specific monoclonal antibodies are lacking. Available evidence suggests that within a class of surface proteins, expression in individual parasites is not strictly mono‐allelic. For example, trypomastigotes can co‐express at least two members of the mucin^183^ and GP85 families.^182^ The finding that a specific mucin‐associated surface protein (MASP) peptide was only expressed in ~5% of parasites indicates that neither is expression totally promiscuous at the protein level.^184^ Mechanisms controlling the expression of parasite surface proteins may therefore vary between gene families or sub‐families. *T. cruzi* may also regulate its antigenic repertoire expression between infection phases and between different host cell types. This requires much deeper analysis because currently there are insufficient data to rule out clonal antigenic variation as a mechanism contributing to perpetual immune evasion.

### 5.2 Parasite dormancy

Many pathogens use dormancy or metabolic quiescence as an immune evasion strategy.^185^, ^186^ At the population level, *T. cruzi* amastigotes can rapidly decrease their replication rate in response to changes in in vitro culture conditions, but this is a function of a longer G_1_ phase rather than exit from the cell cycle.^187^ Individual non‐replicating amastigotes also occur spontaneously in vitro and are less susceptible to the anti‐parasitic drug benznidazole.^21^ The frequency of 4‐day replication arrested in vitro amastigotes has been estimated to be approximately 0.1%‐6%, depending on the parasite strain.^188^ The in vivo relevance of these phenomena remains almost completely unknown and will be hard to establish definitively, not least because neither amastigote DNA/kDNA replication, nor differentiation to constitutively non‐replicating trypomastigotes is synchronized.^22^ Nevertheless, it is reasonable to suspect that slowly replicating or transiently arrested intracellular parasites could have a selective advantage under immunological pressure and play a role in sustaining chronic infections.

### 5.3 Cytokine‐mediated suppression of type 1 responses

*T. cruzi* may continue to benefit from the above‐mentioned conserved negative‐feedback mechanisms that damp down the acute inflammatory response. However, administration of blocking antibodies targeting IL‐10 signalling had no discernible effect on chronic *T. cruzi* infections.^174^ This is in stark contrast to the well‐established role of IL‐10 in promoting chronicity of infections with the related parasite *Leishmania* spp.,^189^ which predominantly infects professional antigen‐presenting cells. Chemical inhibition of the TGF‐beta type I receptor significantly alleviated cardiac pathology and function in chronically infected mice, but this was not associated with any change in heart parasite loads.^190^ It should be noted that parasite loads in the chronic phase are often close to the limit of detection, which means that in these types of intervention experiment it is relatively clear when immunity is compromised, but difficult to conclusively demonstrate a significant enhancement of infection control.

Cytokine gene expression in heart tissue from human patients with severe chronic Chagas cardiomyopathy remains strongly polarized to a type 1 profile.^191^ Type 2 cytokine (IL‐4, IL‐5, IL‐13) expression is reported as undetectable,^191^ and while it is a feature of some animal models, this is apparently not at the expense of IFNγ production.^170^ Interestingly, helminth co‐infection is associated with reduced control of *T. cruzi* in a subset of patients, potentially as a result of a modulation of the cytokine balance.^192^ Overall, cytokine‐mediated suppression of anti‐parasitic type 1 inflammation likely influences the host‐parasite equilibrium and long‐term disease progression, but there is little evidence that it explains *T. cruzi* chronicity.

### 5.4 Immunological exhaustion

T‐cell exhaustion is a feature of many infectious and non‐infectious diseases that involve chronic antigen stimulation and this has been a recent focus of research in the Chagas disease field. Analysis of PBMCs from Chagas patients revealed increased frequencies of CD4^+^ and CD8^+^ T cells expressing exhaustion markers, for example PD‐1^+^, CTLA4^+^, or TIM‐3^+^.^193^, ^194^, ^195^ Experimental studies suggest the development of exhaustion characteristics may be promoted by suboptimal B cell,^152^ IL‐17A^196^ or IL‐10^156^ immune responses. Nevertheless, in chronically infected children and mice, both effector and memory CD8^+^ T cells retain cytotoxic capacity and there is little to no evidence of *functional* exhaustion.^119^, ^152^, ^174^, ^177^, ^197^ Infection chronicity may also promote dysregulation of the Tfh and B‐cell compartments, for example, distinct phenotypes and frequencies of these have been noted between symptomatic and asymptomatic *T. cruzi*‐infected individuals.^198^, ^199^ Whether these alterations reflect a process of B‐cell exhaustion which negatively impacts parasite control remains to be further elucidated. In summary, while deterioration of lymphocyte functional capacity may potentially be associated with progression from asymptomatic to symptomatic disease states, via progressively loosened control of parasite loads, exhaustion does not seem to be a core reason for parasite persistence *per se*.

### 5.5 Local and hyper‐local immune privilege

The realization that long‐term *T. cruzi* infections exhibit an unexpectedly high degree of spatio‐temporal dynamism^4^ indicates that host responses and evasion mechanisms, including those set out above, need to be studied more intensively at the tissue‐specific level. Motile trypomastigotes probably traffic between tissues in both blood and lymph, but there is also evidence that a significant amount of parasite trafficking between tissues may occur inside SLAMF1^+^ myeloid cells, akin to a Trojan horse strategy.^200^ Consequently, parasites from privileged reservoir sites, such as the digestive tract, may seed other, less permissive sites such as the heart, resulting in episodic cycles of re‐invasion and locally sterilizing host responses (Figure 2).^26^ Tissue‐specific variability in permissiveness is consistent with divergent responses observed in different secondary lymphoid organs.^201^ Moreover, when chronically infected mice are immunosuppressed, the infection relapses first in the GI tract and then disseminates to other organs.^29^ Host microbiota may also play a role: its composition can be modulated by *T. cruzi* infection,^202^ but it is not yet known whether this in turn influences anti‐parasite immunity in barrier tissues.

To keep up with the parasite, effector cells must be continually deployed to infection foci in many organs. There appears to be no problem with T‐cell homing and entry into infected tissues,^203^ which is dependent on expression of integrins including VLA‐4 and LFA‐1,^174^, ^204^ and CXCR3 chemokine receptor signalling.^205^ After extravasation though, the distinct microenvironment of each organ potentially drives phenotypic changes to infiltrating cells, and in some cases, the effect may be tolerogenic and incompatible with local sterilization. For example, skeletal muscle bulk CTLs recovered from early chronic phase mice produced less IFNγ and had greatly diminished cytotoxic activity compared to splenic CTLs.^206^ Similar results have been reported for cardiac muscle compared to blood.^203^ Intriguingly, splenic CTLs adoptively transferred from one chronically infected mouse to another retained a high IFNγ response phenotype if they migrated to spleen or lung tissue, but lost it if they migrated to skeletal muscle or liver.^206^ More recently, however, direct ex vivo analysis of parasite‐specific CTLs without antigen re‐stimulation showed cells from chronically infected skeletal muscle tissue had equal or even greater effector capacity (production of IFNγ, TNF‐α, granzyme B) than spleen‐derived cells.^174^ To our knowledge, detailed analysis of CTLs in smooth muscle has yet to be conducted.

There is thus likely to be further compartmentalization of response and evasion dynamics at the intra‐organ level, perhaps even down to the hyper‐local scale of individual infected cells’ microenvironments. For example, the muscular, neuronal and mucosal layers of the GI tract, a key site of *T. cruzi* persistence, have distinct immunological microenvironments that respond differently to *Salmonella* infection.^207^ Recent work has highlighted differences in the cellular composition of perivascular and parenchymal inflammatory infiltrates in *T. cruzi*‐infected skeletal muscle.^171^, ^178^ Large, apparently immunologically invisible parasite nests even occur immediately adjacent to severely inflamed blood vessels, which led these authors to suggest leucocytes might fail to migrate through the parenchyma to infected cells because chemoattractant signalling is too weak in low parasite load settings.^171^ Immune evasion may also operate at the level of physical interaction between T cells and parasite antigen‐presenting cells, for example via manipulation of MHC class I or II expression^107^, ^208^, ^209^, ^210^ or by parasitism of muscle cells, which are poor activators of NF‐κB upon *T. cruzi* infection^76^ and, in the case of skeletal muscle, do not normally express MHC class I.^211^

## 6 CONCLUSIONS

From its origins in ancient South American fauna, *T. cruzi* has spread to diverse mammalian orders across the Americas and become a widespread human pathogen. This reflects a remarkable adaptability to evade mammalian immune responses and maintain enzootic, zoonotic and anthroponotic transmission cycles. As we have set out, this involves sophisticated molecular mechanisms that allow *T. cruzi* to resist innate responses so that in the early stages of infection parasite loads are high and widely disseminated in blood and solid organs. The adaptive immune response, principally effected by CTLs and antibodies, is able to eliminate ~99% of the parasites. The infection then transitions to a permanent chronic phase in which parasites replicate, mainly within muscle cells in a small number of privileged tissues, in a dynamic equilibrium with host responses. The available evidence supports the existence of a complex set of molecular and cellular mediators that firstly, prevent complete sterile clearance at the acute to chronic transition and, secondly, ensure perpetual parasite persistence during the chronic phase. These include parasite‐intrinsic evasion mechanisms, direct and indirect manipulation of host responses, and host‐intrinsic deactivating feedback loops. Further complexity arises from *T. cruzi's* cycling between intra‐ and extracellular parasite forms and its trafficking between different organs, tissues and cells, each with specific immunological microenvironments of variable permissiveness.

Important advances in fundamental aspects of tissue level immunity have yet to be investigated in detail in the Chagas disease field, for example, defining relative contributions of tissue‐resident and inflammatory myeloid cells, innate lymphoid cell populations, tissue‐resident memory T cells and neuro‐immune interactions. Further progress in understanding *T. cruzi*‐host interactions and how they shape Chagas disease pathogenesis is also likely to come from more intensive research at the tissue‐specific and even single cell scale. Some key questions include the following: (a) why do some infected people remain asymptomatic carriers while others progress to life‐threatening disease states? (b) Does active infection support concomitant immunity to second infections? (c) Do drug‐cured patients have protective immunity to re‐infection? (d) Can vaccines be developed that provide sterile protection? (e) To what degree are the proposed mechanisms of immune evasion actually enabling parasite persistence in vivo in different tissues? (f) How do host‐parasite interactions promote or limit *in utero* transmission? The difficulties of answering these questions and addressing the wider challenges in Chagas disease biomedicine are great; however, the massive unmet need for better treatments, prophylaxis and diagnostics requires us to overcome them.

## CONFLICT OF INTEREST

None.

### Peer Review

The peer review history for this article is available at https://publons.com/publon/10.1111/pim.12786.

## Data Availability

Data sharing not applicable to this article as no data sets were generated or analysed during the current study.
